# Notes and News

**Published:** 1901-12-28

**Authors:** 


### Notes and News.

The Committee of Queen Charlotte's Hospital have received an anonymous donation of £200 in aid of their funds.

An entertainment of an excellent character was given to the in-patients of the Cancer Hospital, Fulham Road, on Thursday evening last, arranged by Mr. Fred. C. Everill and friends.

At a meeting of the Court of the Girdlers' Company, recently held at Girdlers' Hall, between £400 and £500 was distributed among the various London hospitals, homes, and other charities.

With the object of preventing the spread of tuberculosis among the American troops engaged in the Philippines, an order has recently been issued from headquarters prohibiting spitting on the floors or walls of offices, barracks, and other buildings under the control of the military authorities.

In the case of the Irish emigrant, Thomas Boden, who was lately refused admission to the United States on the ground that he was suffering from tuberculosis, which was held to be a "loathsome or dangerous disease," appeal was made to the United States Court, where the decision has now been given in favour of the Treasury officials who had ordered his deportation.

At the North London Police Court last week a dealer in foreign postage stamps residing in Islington was charged with having unlawfully exposed himself and carried on his business while suffering from scarlet fever. He had consulted a medical practitioner who, having come to the conclusion that he was suffering from scarlet fever, told him to go home and isolate himself in one room for at least a month. The doctor notified the case, and on the sanitary inspector calling the next day, the patient was found to be out, and this was found to be the case on three successive days. The defendant did not dispute the facts, but said he had his living to get, and, moreover, that he was not sure that he had the disease, as he really was not unwell. He was fined £5 on each summons, or £15 in all, but he seems to have preferred to go to prison for a month.

The directors of the West Point Military Academy, where so many of the United States officers are trained, have recommended that the study of hygiene should be included in the course.

A handsome tablet of bronze has recently been erected in the entrance hall of the Italian Hospital, Queen's Square, in memory of the founder, the inscription on which is as follows:—"To the memory of G. B. Ortelli, Commendatore of the Crown of Italy, Founder of this Hospital and Donor of this land and building."

The National Association for the Prevention of Consumption have issued a letter, in an appendix to which it is stated on the authority of one of his Majesty's inspectors under the Cotton Cloth Factories Act, that as the result of the introduction of improvements in the ventilation of certain factories, a remarkable diminution in the unwholesomeness of the atmosphere has been made. Indeed, the average reduction in the respiratory impurity was upwards of 50 per cent., and in several instances this impurity was brought down to less than one-third of that previously existing.

The report of the committee appointed to investigate the distressing outbreak of tetanus which followed the prophylactic administration of diphtheria antitoxin in St. Louis a little time ago has now been issued. It is to the effect that the incriminated serum was prepared by the Health Department of the City of St. Louis, and that although it was sterile, it contained the toxin of the tetanus bacillus in considerable amount. The facts of the case seem to indicate that the proceedings at the laboratory must have been conducted with considerable carelessness, and that some at least of the serum was issued before it was possible for it to have been properly tested.

This unfortunate occurrence at St. Louis only goes to emphasise what we have more than once urged, that the manufacture and testing of all such biological therapeutic substances as vaccines and antitoxins should be under direct Government control

It would appear from a return presented by the Registrar to the General Medical Council, in regard to the voting at the recent election of direct representatives on that body, that the members of the profession are showing an increasing indisposition to make use of the voting papers sent to them. Of late years the constituency has somewhat increased. In 1896 and 1897 the number of voting papers sent out was almost identical, being 22,577 in the former year, and 22,576 in the latter. This year 23,305 papers were issued, showing an increase of 729 over the number for 1897. The number of voting papers returned in proper time in 1896 was 12,227, and in 1897 was 13,466. On the present occasion only 8,971 were duly returned. Thus, while 54·2 per cent. of the voting papers were returned in 1896 and 59·64 per cent. in 1897, this year only 38·49 per cent. were returned. This does not speak very well for the interest taken by practitioners in direct representation.

